# High-dose radiotherapy is associated with better local control of bone metastasis from hepatocellular carcinoma

**DOI:** 10.18632/oncotarget.14858

**Published:** 2017-01-27

**Authors:** In-Hye Jung, Sang Min Yoon, Jungwon Kwak, Jin-Hong Park, Si Yeol Song, Sang-Wook Lee, Seung Do Ahn, Eun Kyung Choi, Jong Hoon Kim

**Affiliations:** ^1^ Department of Radiation Oncology, Asan Medical Center, University of Ulsan College of Medicine, Seoul, Republic of Korea

**Keywords:** hepatocellular carcinoma, bone metastasis, palliative treatment, radiotherapy, dose-response relationship

## Abstract

We evaluated the pain and radiologic response, time to progression, and dose-response relationship after palliative radiotherapy for bone metastasis from hepatocellular carcinoma. We retrospectively reviewed the medical records of 91 patients between January 2004 and August 2012. The reviewed medical records included data on changes in pain, local tumor progression, and radiologic response evaluated via follow-up images. The radiologic response was assessed based on the Response Evaluation Criteria In Solid Tumors. The pain response was defined according to the International Bone Metastases Consensus Working Party palliative radiotherapy endpoints. Median radiation dose was 40 Gy (range, 20–66 Gy), with various fraction sizes (range, 2.0–6.0 Gy). Pain response rate was 81.4%. During the follow-up periods, radiologic local tumor progression was found in 42 patients (46.2%). The median time to progression was 14.1 months. When the patients were divided into two groups according to their radiation dose (< 55 Gy_10_ vs. ≥ 55 Gy_10_), the pain response rates of the high- and low-dose groups did not differ significantly (*p* = 0.728). However, the radiologic response rate and the time to progression showed significant differences between the two groups (*p* = 0.009 and *p* = 0.018, respectively). With dose escalation, higher radiologic response rates and a longer time to progression were achieved in patients with mass-forming bone metastases from hepatocellular carcinoma.

## INTRODUCTION

Hepatocellular carcinoma (HCC) is the sixth most common cancer and the third leading cause of cancer-related mortality worldwide [1, 2]. Unfortunately, the prognosis of this disease is still poor, with most patients suffering from both chronic hepatitis and frequent tumor recurrence. Moreover, extrahepatic metastasis is common and is being observed more frequently due to improved diagnostic methods and prolonged patient survival [3]. The most frequent extrahepatic metastasis site is the lung, followed by bone, lymph nodes, and adrenal gland. Bone metastasis in HCC occurs in 25–39% of patients with extrahepatic metastasis [3, 4].

Generally, radiotherapy is the treatment of choice for uncomplicated symptomatic bone metastases. Many prospective randomized studies and meta-analyses reported that there were no significant differences in pain control after various radiotherapy regimens; nevertheless, whereas only 2–9% of patients treated with a multi-fraction regimen required repeated radiotherapy, up to 28% of patients treated with a single fraction regimen required repeated radiotherapy [5–9]. In terms of pain relief, most previous studies failed to show a dose-response relationship, but the sites of primary tumors from these studies were mainly prostate, breast, and lung cancers, not HCC [5, 7–10].

Bone metastasis from HCC is characterized by soft-tissue expansion with abundant vascular component [10, 11]. It is thought that a higher radiation dose can result in better local control of these mass-forming bone metastases. According to previous studies, HCC shows a dose-response relationship to radiation, which indicates that total dose is the most important factor in tumor response [12]. In light of this fact, some researchers also studied dose-escalation for bone metastasis from HCC and reported a better radiologic response as well as local control when patients were given higher radiation doses to the metastatic sites [13, 14]. However, only limited data have been reported on the duration of radiologic response after radiotherapy. Therefore, in this study, we evaluated the dose-response relationship, time to progression, and tumor control probability in order to recommend an optimal radiotherapy dose for bone metastasis from HCC.

## RESULTS

### Patient characteristics

The median follow-up duration was 22.8 months (range, 11.9–82.3 months). Of the 91 patients in our study population, 86 died during the follow-up due to HCC progression. The median age at the diagnosis of bone metastasis was 55 years (range, 37–79 years). Twenty-one patients (30.8%) were diagnosed with bone metastasis at the time of diagnosis of HCC. The median duration from the diagnosis of HCC to the diagnosis of bone metastasis was 10.0 months (range, 0–172.2 months). The mean tumor diameter was 4.0 cm (range, 1.5–13.3 cm). A mass-forming tumor was found in 87 patients (95.6%). The most common site of the bone metastases was the spine, followed by the ribs and the pelvis. More detailed patient characteristics are listed in Table 1.

**Table 1:** Patient characteristics

Characteristics	No. of patients (%)			*P*-value
	Total (*n* = 91)	High-dose^a^ (*n* = 45)	Low-dose^b^ (*n* = 46)	
Age (years)				0.683
Median	55	55	54	
Range	37–79	37–72	37–79	
Gender				0.321
Male	80 (87.9)	38 (84.4)	42 (91.3)	
Female	11 (12.1)	7 (15.6)	4 (8.7)	
ECOG PS				0.204
0–1	84 (92.3)	43 (92.6)	41 (89.1)	
2–3	7 (7.7)	2 (4.4)	5 (10.9)	
Child-Pugh class				0.417
A	83 (91.2)	42 (93.3)	41 (89.1)	
B	7 (7.7)	3 (6.7)	4 (8.7)	
C	1 (1.1)	–	1 (2.2)	
Alpha-fetoprotein (ng/mL)				0.322
Median	77.0	101.0	34.9	
Range	1.0–540,277	1.0–470,000	2.0–540,277	
Intrahepatic control				0.372
Yes	67 (73.6)	33 (73.3)	34 (73.9)	
No	24 (26.4)	12 (26.7)	12 (26.1)	
Other extrahepatic metastasis			0.236	
Yes	29 (31.9)	17 (37.8)	12 (26.1)	
No	62 (68.1)	28 (62.2)	34 (73.9)	
Location				< 0.001
Spine	35 (38.5)	6 (13.3)	29 (63.0)	
Rib	30 (33.0)	18 (40.0)	12 (26.1)	
Pelvis	19 (20.9)	15 (33.4)	4 (8.7)	
Etc^c^	7 (7.6)	6 (13.3)	1 (2.2)	
Tumor size (cm)				0.003
Mean diameter	4.0	4.9	3.9	
Range	1.5–13.3	2.0–13.3	1.5–9.0	
Pathologic fracture				0.913
Yes	48 (42.7)	24 (53.3)	24 (52.2)	
No	43 (47.3)	21 (46.7)	22 (47.8)	

*Abbreviations*: ECOG PS, Eastern Cooperative Oncology Group performance status.

^a^High-dose: BED_10_ ≥ 55 Gy_10_

^b^Low-dose: BED_10_ < 55 Gy_10_

^c^The Etc sites included the skull, scapula, clavicle, and sternum.

### Radiotherapy and other treatments

Radiotherapy was planned using 2D (*n* = 16, 17.6%) and 3D conformal radiotherapy (*n* = 74, 81.3%) or intensity-modulated radiotherapy (IMRT) (*n* = 1, 1.1%). The median prescription dose was 40 Gy (range, 20–66 Gy), and the median fraction size was 3 Gy (range, 2–6 Gy). Because the fraction sizes varied among patients, we analyzed the biologically equivalent dose (BED) for correction of different fraction sizes. The median BED of the total irradiation dose was 50.7 Gy (range, 28–85.3 Gy, α/β = 10, Table 2).

**Table 2:** Summary of the prescribed radiation dose

Radiation dose	Median (range)		
	Total (*n* = 91)	High-dose (*n* = 45)	Low-dose (*n* = 46)
Total dose (Gy)	40.0 (20.0–66.0)	50.0 (40.0–66.0)	39.0 (20.0–40.0)
BED_10_	50.7 (28.0–85.3)	62.5 (56.0–85.3)	50.0 (28.0–50.7)
EQD2	42.3 (23.3–71.0)	52.1 (46.7–71.0)	41.7 (23.3–42.3)
Fraction size (Gy)	3.0 (2.0–6.0)	3.0 (2.0–6.0)	3.0 (3.0–4.0)

*Abbreviations*: BED, biologically equivalent dose; EQD2, Equivalent dose in 2 Gy fractions.

The patients were divided into two dose groups depending on their total dose. The patients with a radiation dose higher than 55 Gy BED_10_ were designated as the high-dose group (*n* = 45, 49.5%), and the other patients were designated as the low-dose group (*n* = 46, 50.5%). The detailed patients’ characteristics by dose group are presented in Table 1. There were no significantly different characteristics between the two groups, except for radiotherapy sites and tumor sizes.

Eleven patients (12.1%) were administered with sorafenib before or after radiotherapy as a systemic treatment. Among them, 5 patients belonged to the low-dose group and the others belonged to the high-dose group.

### Pain response

Of the 91 patients, pain response was assessed in 59 patients (64.8%) because 26 patients did not experience pain before radiotherapy, and the change in pain scores could not be assessed in 6 patients owing to the lack of medical records. The median NRS was 3 (range, 1–10). After the radiotherapy, symptomatic pain was reduced in 48 patients (81.4%). The detailed changes in pain score are summarized in Table 3. The pain response rates of the high-dose and low-dose groups did not significantly differ (*p* = 0.73), and these response rates are not significantly different according to the GTV volume (GTV ≤ 26 mm^3^ vs. GTV > 26 mm^3^, *p* = 0.668). However, all of the patients who experienced progressive pain were in the low-dose group (total dose: 20–39 Gy and BED_10_: 28–50.7 Gy).

**Table 3:** Pain responses according to changes in numeric rating scale scores before and after radiotherapy

Response	No. of patients (%) (*n* = 91)	Pre-RT NRS score (mean ± SD)	Post-RT NRS score (mean ± SD)	Change of NRS^a^ (mean ± SD)
CR	24 (26.4)	4.0 *±* 0 2	0.0 *±* 0.0	−4.0 *±* 1.7
PR	24 (26.4)	5.4 *±* 1.9	1.4 *±* 0.6	−4.0 *±* 2.0
SD	7 (7.7)	4.0 *±* 2.0	3.7 *±* 1.4	−0.3 *±* 0.8
PD	4 (4.4)	2.8 *±* 1.3	5.3 *±* 1.7	+2.5 *±* 0.6
N/A	32 (35.1)	–	–	–

*Abbreviations*: NRS, numeric rating scale; CR, complete response; PR, partial response; SD, stable disease; PD, progressive disease; N/A, not assessed.

^a^*P*-values of the changes of NRS using one-way ANOVA : overall, *p* < 0.001; CR vs. PR, *p* = 1.000; CR vs. SD, *p* < 0.001; CR vs. PD, *p* < 0.001; PR vs. SD, *p* < 0.001; PR vs. PD, *p* < 0.001; SD vs. PD, *p* = 0.063.

### Radiologic response and time to progression

The radiologic responses were evaluated in all of the patients. The radiologic response rate after radiotherapy was 53.9% and the best radiologic response rates are summarized in Table 4. The radiologic responses of the two dose groups differed significantly (*p* = 0.009). The median time to the best radiologic response was 4.9 months (range, 0.7–15.3 months). In univariate and multivariate analyses of prognostic factors for radiologic response, location of bone metastasis and chemotherapy were statistically significant factors (Table 5).

**Table 4:** Best radiologic response by dose group

Response	No. of patients (%)			*P*-value
	Total (*n* = 91)	High-dose (*n* = 45)	Low-dose (*n* = 46)	
CR	5 (5.5)	4 (8.9)	1 (2.2)	0.009
PR	44 (48.3)	26 (57.8)	18 (39.1)	
SD	38 (41.8)	14 (31.1)	24 (52.2)	
PD	4 (4.4)	1 (2.2)	3 (6.5)	

*Abbreviations*: CR, complete response; PR, partial response; SD, stable disease; PD, progressive disease.

**Table 5:** Prognostic factors for radiologic response

Variable	No. of patients (%)	Univariate analysis		Multivariate analysis	
		C. OR (95% CI)	*P*-value	A. OR (95% CI)	*P*-value
Gender		1.033 (0.291–3.661)	0.960		
Male	80 (87.9)				
Female	11 (12.1)				
Age		0.720 (0.314–1.649)	0.437		
≤ 55 years	48 (52.7)				
> 55 years	43 (47.3)				
Viral etiology		0.450 (0.171–1.183)	0.105		
HBsAg (+)	68 (74.7)				
HBsAg (−)	23 (25.3)				
ECOG PS		0.315 (0.058–1.716)	0.182		
0–1	84 (92.3)				
2–3	7 (7.7)				
Child-Pugh class		1.184 (0.227–5.057)	0.819		
A	83 (91.2)				
B, C	8 (8.8)				
Alpha–fetoprotein		1.748 (0.673–4.540)	0.251		
≤ 7.5 ng/mL	23 (25.3)				
> 7.5 ng/mL	68 (74.7)				
Other metastasis		1.082 (0.446–2.622)	0.862		
No	62 (68.1)				
Yes	29 (31.9)				
Location		4.606 (1.858–11.416)	0.001	5.339 (2.056–13.863)	0.001
Spine	35 (38.5)				
Non-spine	56 (61.5)				
Chemotherapy		0.156 (0.032–0.769)	0.022	0.119 (0.022–0.631)	0.012
No	80 (87.9)				
Yes	11 (12.1)				
GTV volume		1.243 (0.545–2.839)	0.605		
≤ 26 mm^3^	45 (49.5)				
> 26 mm^3^	46 (50.5)				

*Abbreviations*: C. OR, crude odds ratio; CI, confidence interval; A. OR, adjusted odds ratio; HBsAg, hepatitis B surface antigen; ECOG PS, Eastern Cooperative Oncology Group performance status; GTV, gross tumor volume.

Local tumor progression was observed in 42 patients (46.2%). In the high-dose group, 15 patients (33.3%) showed local progression, whereas in the low-dose group, 27 patients (58.7%) experienced local tumor progression (*p* = 0.015).

**Figure 1:**
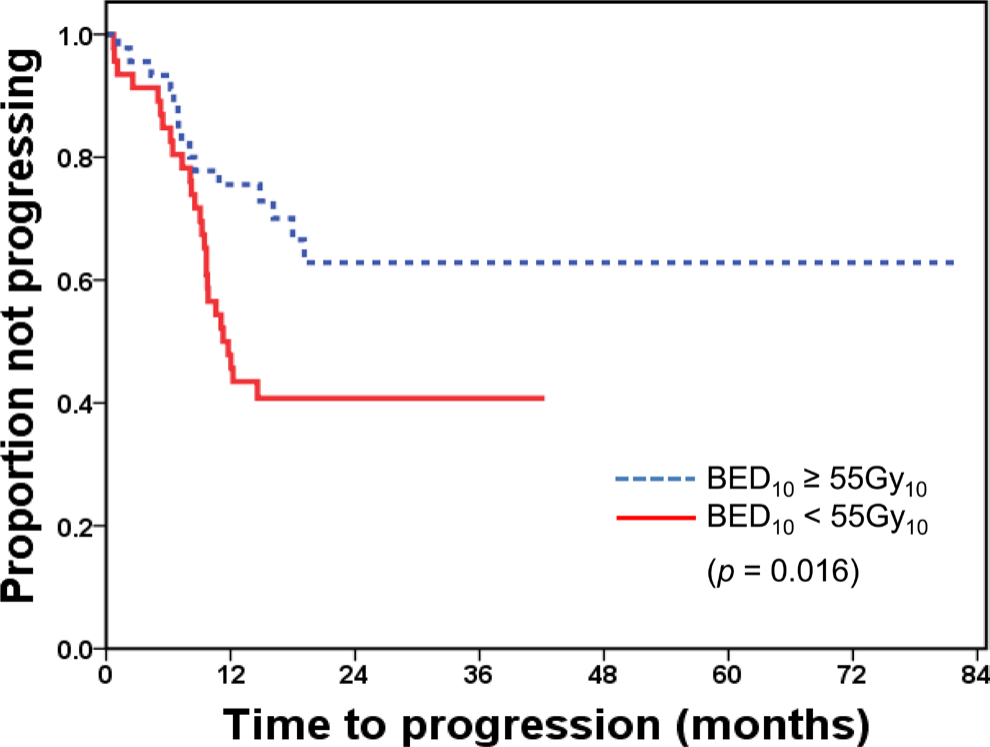
Time to progression after radiotherapy by dose group BED, biologically equivalent dose

The median time to progression for all patients was 14.1 months (range, 0.7–82.0 months). The time to progression significantly differed between the dose groups. The median time to progression in the high-dose group was 16.8 months (range, 1.2–82.0 months), and in the low-dose group, 11.3 months (range, 0.7–42.3 months) (*p* = 0.016) (Figure 1).

**Figure 2:**
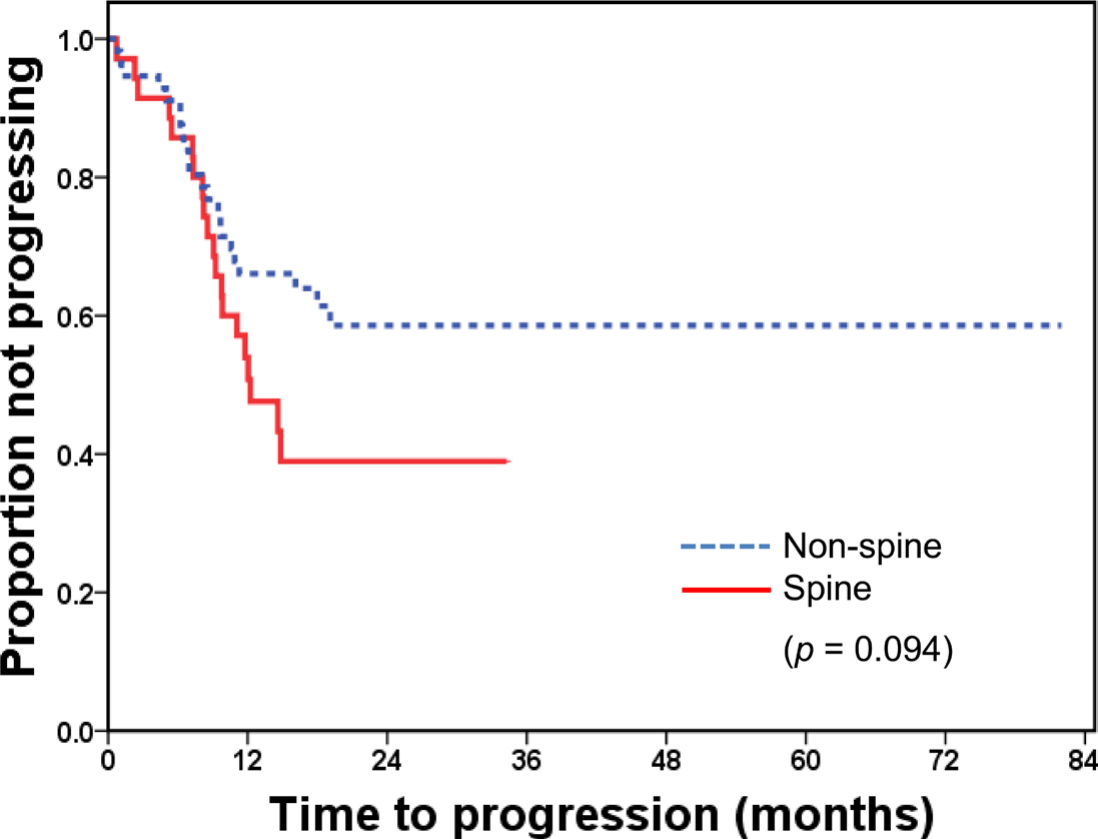
Time to progression after radiotherapy by the site of bone metastasis

The radiologic response rates and the median time to progression did not differ significantly by GTV volume (GTV ≤ 26 mm^3^ vs. GTV > 26 mm^3^, *p* = 0.284, *p* = 0.317, respectively). However, the relationship between radiologic response and location of tumors was statistically significant (Table 5), and time to progression was marginally significant according to the location of bone metastasis (Figure 2).

### Tumor control probability

**Figure 3:**
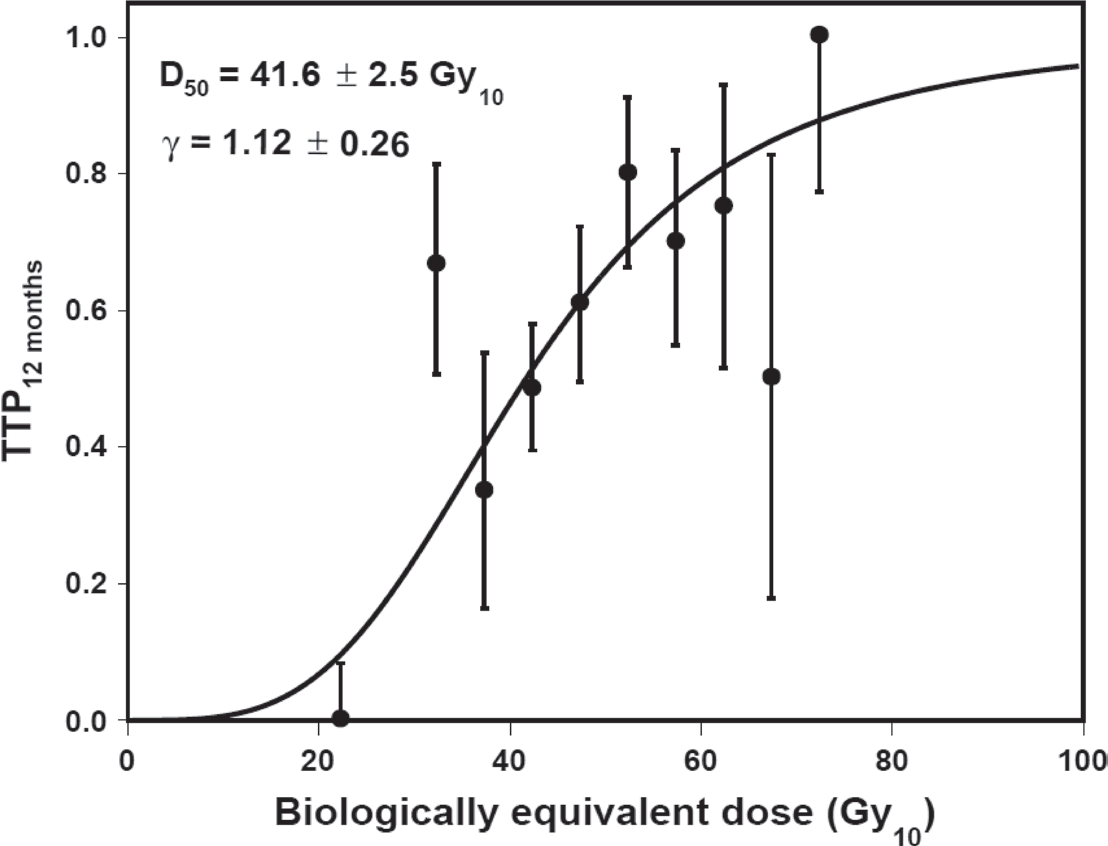
Tumor control probability 12 months after radiotherapy TTP, time to progression

Figure 3 shows the TCP of the total radiation dose (BED_10_) versus the progression-free survival 12 months after the radiotherapy for all patients (fitted parameters: γ = 1.12, D_50_ = 41.6 Gy_10_, and 95% CI, 39.1–44.1 Gy_10_). According to this TCP curve, 52.8 Gy_10_ provides a 70% local control rate 12 months after radiotherapy.

### Toxicities

The incidence of treatment-related toxicities did not differ significantly between the dose groups. Grade 4 or 5 toxicities were not observed in either group. The most common toxicities were Grade 1 anemia and thrombocytopenia. Grade hematologic 3 toxicities did occur. The details of the toxicities are shown in Table 6. Considering that many of the patients had poor liver function, other treatment modalities such as chemotherapy might have influenced their hematopoietic function. In patients with Grade 3 hematologic toxicities, 16 (69.6%) underwent radiotherapy 1–2 months after trans-arterial chemoembolization. Grade 3 hematologic complications associated purely with radiotherapy were detected in 3 patients. Radiation-induced myelopathy and gastrointestinal bleeding were not observed during the follow-up period.

**Table 6:** Acute and late toxicities by dose group

Toxicities	Grade	No. of patients (%)			*P*-value
		Total (*n* = 91)	High-dose (*n* = 45)	Low-dose (*n* = 46)	
Acute					
GI	0	54 (59.3)	32 (71.1)	22 (47.8)	0.001
(anorexia, nausea, vomiting)	1	18 (19.8)	8 (17.8)	10 (21.7)	
	2	6 (6.6)	4 (8.9)	2 (4.4)	
	3	–	–	–	
	N/A	13 (14.3)	1 (2.2)	12 (26.1)	
Hematologic	0	6 (6.6)	3 (6.7)	3 (6.5)	0.174
	1	28 (30.8)	14 (31.1)	14 (30.4)	
	2	34 (37.3)	21 (46.7)	13 (28.3)	
	3	22 (24.2)	7 (15.5)	15 (32.6)	
	N/A	1 (1.1)	0 (0)	1 (2.2)	
Late					
Myelopathy	0	91 (100)	45 (100)	46 (100)	0.179

*Abbreviations*: N/A, not assessed.

## DISCUSSION

The incidence of bone metastasis from HCC has been reported to be up to 40% [3]. Bone metastases often cause a great deal of discomfort such as pain or associated neurologic symptoms. For a selected group of patients with pathologic fractures or spinal cord compression with progressive symptoms, surgery is a better treatment modality because it quickly relieves neurologic symptoms. However, radiotherapy is generally the treatment of choice for uncomplicated bone metastases due to its non-invasiveness and convenience.

Previously, many randomized controlled trials have been conducted to identify an adequate dose for palliation of bone metastasis [5–7, 9, 19]. These trials have demonstrated the equivalence of multiple- and single-fraction radiotherapy schedules despite different inclusion criteria and primary end-points. Meta-analyses have also reported similar overall pain response rates of about 60% regardless of radiotherapy fractionation schedule [20–22]. The Radiation Therapy Oncology Group (RTOG) reported that they could not confirm dose-response relationships in bone metastasis despite an increased fraction size in 2011 [8]. However, the primary organs in the RTOG reports were mostly the prostate, lung, and breast; therefore, the aforementioned results could not be directly applied to other types of bone metastasis such as mass-forming metastasis. Unlike other forms of metastasis, bone metastases from HCC are usually mass-forming types with invasion of surrounding soft tissues [23, 24]. These features suggest that a higher radiation dose may be necessary to achieve better outcomes, similar to primary HCC which has been reported to have a dose-response relationship with radiation [10, 12].

A few recent studies have reported the dose-response relationships of radiation and bone metastasis from HCC. Choi et al. showed that an elevated total irradiation dose could improve local control in spinal metastases from HCC. Patients in the higher BED (over 56 Gy_10_) group showed that the local control rate of bone metastasis was increased compared to the lower-dose group [14]. Kim et al. also reported significantly different radiologic response rates among different dose groups. In the high-dose group (> 39 Gy_10_), the radiologic response was reported as 89.8%; in the low-dose group, it was 66.3% [25] (Table 7). However, these studies did not report the time to progression after radiotherapy by dose group. The time to progression is an important outcome for patients with bone metastases with regard to their quality of life or need for further treatment such as re-irradiation. In the present study, the high-dose group showed a significantly longer time to progression than the low-dose group. Moreover, according to the TCP analysis, the 52.8 Gy_10_ dose at the bone metastatic site brought about a 70% radiologic progression-free rate 12 months after radiotherapy. These findings suggest that a dose prescription of more than 55 Gy_10_ should be considered for radiotherapy in patients with bone metastasis from HCC, especially for those who are expected to live for more than 1 year.

**Table 7:** Summary of studies on dose escalations in bone metastasis from HCC

Study, year	No. of patients (No. of lesions)	F/U (months) Median (range)	RT dose (Gy) Median (range)	Local failure	Comments
Choi et al., 2014	30	5.6	48.0 (21.0–51.0)	(*p* = 0.019)	
	(42)	(–)	BED_10_ ≤ 56.0 Gy_10_	5/10	–
			BED_10_ > 56.0 Gy_10_	1/20	
Kim et al., 2011	103	6	30.0 (8.0–45.0)	(*p* = 0.02)	
	(223)	(0-46)	BED_10_ < 39.0 Gy_10_	34/101	–
			BED_10_ ≥ 39.0 Gy_10_	5/49	
Current study	91	22.8	40.0 (20.0–66.0)	(*p* = 0.015)	TTP (mo) (*p* = 0.016)
	(91)	(11.9–82.3)	BED_10_ < 55.0 Gy_10_	27/46	11.3
			BED_10_ ≥ 55.0 Gy_10_	15/45	16.8

*Abbreviations*: HCC, hepatocellular carcinoma; BED, biologically equivalent dose; TTP, time to progression.

We observed that the location of bone metastasis was a statistically significant factor for radiologic response. According to the analysis, time to progression was longer in non-spine metastasis that in spine metastasis. It can be assumed that the spinal cord is a major dose-limiting organ in case of bone metastasis in spine, since it is difficult to deliver a higher radiation dose in this region compared to other bone metastatic sites. Stereotactic body radiotherapy (SBRT) for spine metastasis is increasingly used given recent technical advances in radiotherapy technique; the highly sophisticated treatment approach should be considered in appropriate patients. Another significant factor for radiologic response is the use of chemotherapy on binary logistic regression. However, the adjusted odds ratio of systemic chemotherapy was 0.119, which might be interpreted as chemotherapy having a negative effect on radiologic response. Nevertheless, due to the imbalance in number of patients per group (only 11 patients received chemotherapy), it is difficult to accurately define the actual clinical meaning of the relationship between chemotherapy and radiologic response for bone metastasis after radiotherapy. Further studies are necessary to clarify the relationship between these two treatment modalities for bone metastasis

This study had several limitations. First, we selected patients who were followed up for at least a year after radiotherapy. We understand that this could act as a significant confounding factor; however, to achieve the specific purpose of this study, we need to restrict the participant pool to those who had long-term imaging data in order to evaluate their time to progression and TCP so that we could develop recommendations for the optimal radiation dose in this clinical setting. Second, this study was a retrospective study, so it is possible that some data have been left out or the patients’ symptomatic improvement or progression have been underestimated. Third, even though we used BED calculation in order to compensate for various dose schemes, some heterogeneity remained in the planning methods following time from 2D to IMRT, which could be another confounding factor. Despite these limitations, we believe that our data can provide useful information on treating patients with bone metastasis, especially if HCC is the primary tumor. According to our data, for patients with a favorable prognosis, the application of a higher dose should be considered to prevent tumor progression during the follow-up period and to reduce the probability of re-irradiation at the same sites. On the other hand, for patients with a poor prognosis, we assume that low-dose and short-course irradiation is sufficient for short-term symptom palliation.

In conclusion, palliative radiotherapy for bone metastasis from HCC showed a dose-response relationship for the radiologic response rate and the time to progression. If a proper plan can exclude radiosensitive organs below the tolerance dose level using more sophisticated radiotherapy techniques such as SBRT or IMRT, high-dose irradiation (total BED larger than 55 Gy_10_) might be helpful for patients with a good prognosis. However, a prospective study is still necessary to confirm the appropriate radiation dose for bone metastasis from HCC.

## MATERIALS AND METHODS

### Patients

From January 2004 to August 2012, 675 patients underwent palliative radiotherapy for bone metastasis from HCC at Asan Medical Center according to the database registry. Among these patients, we selected patients that met our research purpose using the following inclusion criteria: (1) patients who completed scheduled radiotherapy (50 patients were excluded owing to incomplete radiotherapy), (2) patients that were treated with multi-fraction radiotherapy regimens (8 patients were excluded), and (3) patients that had more than a year of follow-up with computed tomography (CT) or magnetic resonance imaging (MRI) of the treated bone metastatic sites (526 patients were excluded). We limited the study to patients who had long-term follow-up images in order to evaluate the response duration and time to progression after radiotherapy with these serial images. Therefore, 91 patients were included and we reviewed their medical records and imaging work-ups (CT, MRI, or positron emission tomography (PET)).

HCC was diagnosed based on pathologic confirmation or a characteristic tumor appearance on at least two imaging studies (including dynamic CT scans, dynamic enhanced MRI scans, and angiograms) and the presence of risk factors such as infection with hepatitis B or hepatitis C virus, and/or cirrhosis [15]. Bone metastases were diagnosed with imaging studies such as CT, MRI, PET, or bone scans, with histologic confirmation in some patients. A mass-forming metastasis was defined as an extra-osseous soft tissue mass with a clear boundary outside the bone [14].

### Radiotherapy

Indications for external beam radiotherapy were symptomatic bone metastasis, spinal cord compression, high risk of pathologic fracture, or prophylactic treatment to sustain bone stability without development of the symptoms. Except for 16 patients (17.6%) that were treated with two-dimensional treatment planning, three-dimensional (3D) CT simulation was performed under free breathing with or without contrast media using a 16-slice CT (LightSpeed RT 16; GE Healthcare, Waukesha, WI, USA). Immobilizers such as a vacuum cushion were also used during the simulation to enhance reproducibility and accuracy. Gross tumor volume (GTV) was defined as the enhancing mass and osteolytic/osteoblastic changes on involved bone in planning CT, which matched the lesion on the diagnostic images. Clinical target volume (CTV) included the GTV and the involved bony structures extended by 2 to 5 cm from the GTV to encompass the microscopic infiltrations of involved bone marrow according to the physicians’ decisions. Planning target volume (PTV) was expanded 7 to 10 mm from the CTV. Radiotherapy planning was conducted using a 3D radiotherapy planning system (Eclipse; Varian, Palo Alto, CA, USA). Radiotherapy was delivered by 6- or 15-MV X-rays from a linear accelerator (Varian, Palo Alto, CA). The total irradiation dose and fraction size were determined by adjacent organs, such as the spinal cord, stomach, and small or large bowel, in consideration of minimizing the radiotherapy-induced toxicity.

### Evaluation

Most patients were regularly checked using dynamic contrast enhancing CT or MRI at 2- to 3-month intervals to evaluate the primary intrahepatic tumor status. After radiotherapy, imaging work-ups for treated sites were performed at 1- to 3-month intervals. Additional examinations including simple radiography, bone scans, CT/MRI for other sites, or PET/CT were indicated according to the patients’ symptoms. Using the Response Evaluation Criteria In Solid Tumors (RECIST) [16], we assessed the changes in the size of the mass-forming bone metastases and reported the best response after radiotherapy. In-field progression was defined as a 20% or higher increase in the largest diameter than in the best response status. The pain response was defined by referring to the guidelines of the International Bone Metastases Consensus Working Party palliative radiotherapy endpoints [5, 14, 17]. A complete response (CR) was defined as no pain after radiotherapy, partial response (PR) as a reduced numeric pain rating scale (NRS) score of more than 2 points, progressive disease as a disease with a more than 2-point higher pain score after radiotherapy, and stable disease (SD) as a status of neither PR nor PD. Adverse events related to radiotherapy were graded according to the Common Terminology Criteria for Adverse Events (CTCAE version 4.2).

### Statistical analysis

The patients were divided into two groups according to their total irradiation dose for bone metastasis. Because the mean BED_10_ value of all the patients was 54.8 Gy_10_, we used 55 Gy_10_ as the cut-off point. Therefore, patients irradiated with a dose higher than 55 Gy BED_10_ were assigned to the high-dose group and the other patients were assigned to the low-dose group. The treatment responses and the times to the best radiologic response of the high-dose and the low-dose groups were compared using Student's *t-test* or a χ^2^ test. A one-way analysis of variance was utilized to examine the relationship between the changes of NRS scores and pain response.

The time to progression was defined as the period from the start date of the radiotherapy to the date of progression of the disease at an imaging workup, or to the date of symptomatic aggravation, or to the date of the last follow-up. The time to the best radiologic response was calculated from the start date of the radiotherapy to the date of the imaging workup that showed the best response. The times to progression of the two groups were calculated using the Kaplan-Meier method and compared through a log-rank test. *P-value* < 0.05 was considered statistically significant. Prognostic factors for radiologic response were analyzed using binary logistic regression. Variables with a *p-value* ≤ 0.1 in univariate analyses were included in a multivariable logistic regression model. The final model for response was determined by backward stepwise elimination procedures. All of the statistical analyses were performed using the Statistical Package for Social Science version 21 (SPSS Inc., Chicago, IL, USA).

The tumor control probability (TCP) was defined as the end-point of the control of tumor progression at 12 months after the radiotherapy. The X-axis represents the prescribed total dose, the biologically equivalent dose (BED), and the Y-axis represents the probability of tumor control. The TCP for each bin was calculated using the Kaplan-Meier method. The quantification of the dose response to the tumor was estimated using a logistic model as follows:

$$\text{TCP}=\frac{1}{{\left(1+\frac{{\text{D}}_{50}}{\text{D}}\right)}^{4/\gamma }}$$

(D: total dose; γ: slope of the curve; and D_50_: dose that achieves a TCP of 50% for the prescribed dose)

D_50_ and γ were estimated by a logit function. The 95% confidence intervals (CIs) were calculated using the probability density function of the normal distribution. MATLAB R2011b was used for the formula and calculations [18].
